# Cerebral Venous Thrombosis Involving the Vein of Labbé and Left Sigmoid Sinus in a Patient With Immune Thrombocytopenia Treated With Romiplostim: A Case Report and Literature Review

**DOI:** 10.7759/cureus.107599

**Published:** 2026-04-23

**Authors:** Carlos Berrocal, Isabella Libreros Mejia, Gustavo Ramos Burbano, Fernando Araque Villaquirán, Andrés Parra Varela, Mariana Caballero Lagares, Elvis Mauricio Pérez Lafont, Víctor Alonso Carrascal López

**Affiliations:** 1 Internal Medicine, Universidad del Valle, Cali, COL; 2 General Medicine, Universidad Libre, Cali, COL; 3 Internal Medicine, Universidad Libre, Cali, COL; 4 Neurology, Clinica Diagnóstico Médico Especializado (DIME), Cali, COL; 5 Radiology/Internal Medicine, Universidad del Valle, Cali, COL; 6 General Medicine, Hospital Universitario San Vicente de Paúl, Medellín, COL; 7 General Medicine, Universidad del Sinú, Montería, COL; 8 General Medicine, Empresa Social del Estado (ESE) Vida Sinú, Montería, COL; 9 General Medicine, Fundación Amigos de la Salud, Montería, COL

**Keywords:** anticoagulants, prosopagnosia, thrombocytopenia, thrombopoietin receptor agonist (tpo-ra), venous thrombosis (ijv)

## Abstract

Cerebral venous thrombosis is an uncommon but potentially serious condition primarily affecting young women. Thrombosis of the vein of Labbé is a rare site of thrombosis linked to hemorrhagic venous infarction. Thrombotic events have been reported in patients with chronic primary immune thrombocytopenia (ITP) treated with thrombopoietin receptor agonists (TPO-RAs).

A 33-year-old woman with chronic ITP, treated with romiplostim for five years, presented with a progressively worsening occipital headache and a generalized seizure. Computed tomography showed a left parietotemporal intraparenchymal hemorrhage. Magnetic resonance imaging and venography confirmed left sigmoid sinus thrombosis and a hemorrhagic venous infarction caused by thrombosis of the vein of Labbé, along with signs indicating a previous superior sagittal sinus thrombosis. Romiplostim was discontinued after the event, and anticoagulation, along with corticosteroids, was started due to a decline in platelet count. Common causes of cerebral venous thrombosis were investigated and ruled out. The patient was discharged asymptomatic on hospital day 12 and was seen for outpatient follow-up.

This case highlights a rare presentation of Labbé vein thrombosis in the setting of long-term use of TPO-RAs and supports a possible association.

## Introduction

Cerebral venous thrombosis (CVT) is an uncommon yet potentially severe condition, representing about 0.5-1% of all strokes [1], and it predominantly affects young adults, especially women. It results from thrombosis of the cerebral venous system, which can impair venous drainage and lead to hemorrhagic infarction. Thrombosis of the vein of Labbé (LVT), a cortical vein draining the temporal lobe into the transverse sinus, is a particularly rare site of thrombosis, usually resulting from thrombotic extension from the transverse and sigmoid sinuses, and carries a significant risk of hemorrhagic venous infarction in the temporal lobe [2,3].

Among established acquired prothrombotic risk factors are autoimmune disorders, inflammatory states, pregnancy, and malignancies, while the use of thrombopoietin receptor agonists (TPO-RAs) in patients with immune thrombocytopenia (ITP) has emerged as a potential but not fully established association. We report the case of a young woman with chronic ITP on long-term romiplostim who developed LVT with subsequent hemorrhagic venous infarction. In this patient, common causes of CVT were excluded, supporting a possible association with TPO-RA therapy and highlighting the importance of recognizing this potential risk in clinical practice.

## Case presentation

A 33-year-old woman with a history of chronic ITP, reportedly managed with weekly subcutaneous romiplostim (250 mcg) for approximately 2.5 years, presented to the emergency department. Available records suggest a treatment-dependent disease course, with adequate platelet control while on romiplostim and recurrence of thrombocytopenia during treatment interruptions. Notably, a prior episode in September 2021 documented gingival bleeding associated with thrombocytopenia following the discontinuation of romiplostim, requiring treatment with intravenous methylprednisolone and subsequent oral prednisone, supporting the diagnosis of chronic ITP.

Her neurological history was notable for a seizure disorder diagnosed approximately five years earlier, previously treated with valproic acid, and a three-year history of chronic headache. Antiepileptic therapy had been discontinued approximately five years prior, following a period of seizure remission; however, it is unclear whether this was medically indicated or patient-initiated, and details regarding the initial etiology, diagnostic workup, and headache characterization were not fully available due to incomplete medical records.

She was admitted due to the exacerbation of a headache of one week's duration, predominantly occipital, followed by a generalized tonic-clonic seizure associated with a fall, resulting in a moderate traumatic brain injury.

On physical examination, vital signs were within normal limits. Neurological assessment revealed an alert patient who was partially disoriented to time, with evident inattention and deficits in higher cortical functions, including impaired calculation, abstraction, and recent memory. Language was preserved, and cranial nerve examination was unremarkable. Motor examination showed normal muscle strength and tone, with no focal deficits. Sensory examination was notable for digital agnosia and impaired body schema recognition (asomatognosia). Coordination and cerebellar function were intact. Deep tendon reflexes were normal, with no Babinski sign. No meningeal signs were present.

**Figure 1:**
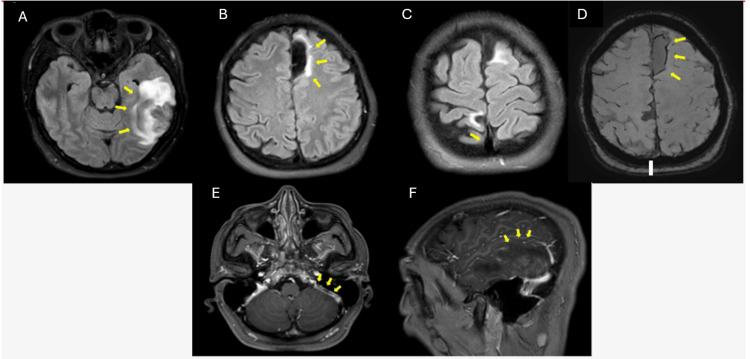
Brain MRI findings (A) Axial FLAIR sequence demonstrating vasogenic edema predominantly in the left temporal lobe, with associated hemorrhagic components (yellow arrows), consistent with hemorrhagic venous infarction. (B-C) Areas of encephalomalacia and gliosis involving the left frontal lobe (B) and the right postcentral gyrus (C) (yellow arrows), suggestive of prior ischemic or hemorrhagic events. (D) Axial SWI showing superficial siderosis over the left frontal convexity, indicative of a previous hemorrhagic insult. (E-F) Post-contrast 3D MRI (axial and sagittal views): (E) filling defect within the left sigmoid sinus (arrow), consistent with subacute venous thrombosis, and (F) absence of flow in the vein of Labbé and its cortical tributaries (yellow arrows), confirming cortical venous involvement. Overall, these findings are consistent with extensive cerebral venous thrombosis involving the left sigmoid sinus and the vein of Labbé, associated with a hemorrhagic venous infarction in the left temporal lobe. MRI: magnetic resonance imaging; FLAIR: fluid-attenuated inversion recovery; SWI: susceptibility-weighted imaging

A brain computed tomography (CT) scan revealed multiple punctate and confluent intraparenchymal hemorrhagic foci in a cortical-subcortical distribution, predominantly involving the left temporal lobe, with extension toward the parietal region. These findings were associated with surrounding cytotoxic edema, without significant mass effect. Given the atypical distribution and multifocal pattern of intracranial hemorrhage, CVT was suspected. Brain magnetic resonance imaging (MRI), magnetic resonance angiography (MRA), and contrast-enhanced venography were subsequently performed. These studies demonstrated a filling defect in the left sigmoid sinus consistent with subacute thrombosis, along with the absence of flow in the vein of Labbé and its tributaries, supporting the diagnosis of cortical venous thrombosis. In addition, there was imaging evidence of prior partially recanalized thrombosis of the superior sagittal sinus. Parenchymal findings were consistent with hemorrhagic venous infarction predominantly affecting the left temporal lobe, in keeping with the drainage territory of the vein of Labbé (Figure 1).

Further investigations were conducted to identify a potential embolic source and thrombophilia (Table 1). Cardiac evaluation, including transthoracic echocardiography and 24-hour Holter monitoring, was unremarkable. Initial thrombophilia screening, including anticardiolipin antibodies, was negative. Autoimmune testing revealed high-titer antinuclear antibodies (ANA), while extractable nuclear antigen (ENA) screening was negative. Human immunodeficiency virus (HIV) testing was negative.

**Table 1:** Laboratory findings BUN: blood urea nitrogen; INR: international normalized ratio; aPTT: activated partial thromboplastin time; ANA: antinuclear antibodies; C3 and C4: complement components 3 and 4; HIV: human immunodeficiency virus; ENA: extractable nuclear antigen antibodies; IgG: immunoglobulin G; IgM: immunoglobulin M; COI: cut-off index

Test	Result	Reference range
Leukocytes (cells/µL)	8,330	4,000-10,000
Hemoglobin (g/dL)	11.4	12-16
Hematocrit (%)	35.4	36-46
Platelets (cells/µL)	220,000	150,000-450,000
Creatinine (mg/dL)	1.07	0.5-1.1
BUN (mg/dL)	16.6	7-20
INR	Normal	0.8-1.2
aPTT (seconds)	28.8	25-35
ANA	1:640 fine speckled nuclear pattern (AC-4)	-
ENA (total)	Negative	-
C3 (mg/dL)	125	90-180
C4 (mg/dL)	31.94	10-40
HIV (COI)	0.09	Negative: <1.0
Homocysteine (µmol/L)	7.96	5-15
Anticardiolipin antibodies (IgG/IgM) (GPL/MPL U/mL)	<10	<10-15

The most likely contributing factors were the history of ITP and the use of romiplostim. The TPO-RA was discontinued, and therapeutic anticoagulation with low-molecular-weight heparin (enoxaparin 70 mg subcutaneously every 12 hours) was initiated and later transitioned to apixaban (5 mg orally twice daily).

During hospitalization, a progressive decline in platelet count was observed, decreasing from 220,000 cells/µL at admission (day 1) to 116,000 cells/µL on day 6, 82,000 cells/µL on day 9, and 65,000 cells/µL on day 11. Given the downward trend, oral prednisolone (50 mg daily for two weeks) was initiated on day 11 of hospitalization. Platelet counts subsequently reached a nadir of 51,000 cells/µL on day 14. Despite this decline, levels remained above the safety threshold of 30,000 cells/µL, allowing the continuation of anticoagulation.

The patient was discharged after 12 days of hospitalization, asymptomatic, on oral prednisolone and apixaban, with outpatient follow-up planned to determine the definitive management of ITP.

## Discussion

LVT is a rare form of CVT involving the vein of Labbé, also known as the inferior anastomotic vein. This cortical vein connects the superficial sylvian venous system with the transverse sinus and drains the lateral surface of the temporal lobe through an oblique course along its inferolateral aspect [2]. This region is functionally associated with language, memory, auditory processing, and higher cognitive functions.

LVT is commonly associated with significant thrombotic extension from the transverse sinus, frequently involving the sigmoid sinus, leading to the complete occlusion of the ipsilateral venous drainage system [3]. Its prevalence in the context of transverse sinus thrombosis is approximately 15%, based on MRI studies in adult cohorts, predominantly affecting young individuals, with a mean age of presentation around 33 years and a female predominance [3]. This entity is clinically relevant due to the high risk of venous infarction, often with hemorrhagic transformation, which explains the variability in neurological manifestations [4].

The pathophysiology of CVT is classically described by Virchow's triad: venous stasis, endothelial injury, and hypercoagulability [5]. In the cerebral circulation, venous stasis may result from impaired outflow secondary to thrombosis of adjacent dural sinuses, such as the transverse sinus, increasing the susceptibility of cortical veins, including the vein of Labbé, to occlusion [4]. Thrombus propagation is further promoted by persistent stasis and hypercoagulability, which may be either hereditary (thrombophilia) or acquired (e.g., malignancy, pregnancy, or oral contraceptive use) [6].

In our patient, an extensive workup excluded common etiologies, with chronic ITP and the use of TPO-RAs identified as the only potential prothrombotic factors, supporting a likely multifactorial mechanism.

In our literature review, we identified 12 reported cases of CVT in patients with ITP receiving TPO-RAs (Table 2) [7-17]. Of these, 11 occurred in women; 10 were associated with eltrombopag, and only one was associated with romiplostim. Notably, thrombotic events occurred in patients with normal or moderately elevated platelet counts, suggesting that thrombosis is not exclusively dependent on severe thrombocytosis. Events were observed early both after treatment initiation and after prolonged exposure. The most frequent locations involved major dural venous sinuses, including the superior sagittal, transverse, and sigmoid sinuses. Although most cases had favorable outcomes following anticoagulation and drug discontinuation, TPO-RA-associated CVT can be potentially fatal, as evidenced by one reported death [14]. Taken together, these findings suggest that TPO-RA-associated CVT may occur across a wide range of clinical scenarios, including in the absence of marked thrombocytosis and after prolonged drug exposure, while predominantly affecting major dural sinuses. In contrast to previously reported cases, our patient exhibited thrombosis involving the vein of Labbé, a rare cortical venous location, along with evidence of prior partially recanalized superior sagittal sinus thrombosis, suggesting a possible recurrent or subclinical thrombotic process. Furthermore, the event occurred during prolonged exposure to romiplostim and in the absence of significant thrombocytosis or alternative etiologies, highlighting a broader and potentially underrecognized spectrum of TPO-RA-associated thrombotic risk.

**Table 2:** Comparison of the reported cases of CVT associated with TPO-RA in patients with ITP ITP: immune thrombocytopenia; TPO-RA: thrombopoietin receptor agonist; OCP: oral contraceptives; ANA: antinuclear antibodies; IgM: immunoglobulin M; APLA: antiphospholipid antibodies; CVT: cerebral venous thrombosis; LMWH: low-molecular-weight heparin; IV: intravenous; N/A: not available

Author, year, country	Sex/age	Initial presentation	ITP duration	TPO-RA (dose)	TPO-RA duration	OCP use	Platelets (/mm³)	Autoimmunity/thrombophilia	Thrombosis site	Treatment and outcome
Mulla et al. [7], 2014, USA	F/55	Headache, nausea, vomiting	18 years	Eltrombopag (50 mg)	13 days (3 days after dose increase)	No	124,000	Negative	Right transverse and sigmoid sinuses and internal jugular vein	Heparin followed by warfarin. Resolution
Ho et al. [8], 2015, Australia	F/45	Occipital headache, papilledema, ptosis	Chronic refractory	Romiplostim (6 mcg/kg)	6 weeks (6 doses)	No	31,000-35,000	Elevated IgM anticardiolipin (110 U)	Extensive CVT involving major sinuses	Heparin followed by warfarin. Complete recovery
Nambiar et al. [9], 2016, India	F/36	Menorrhagia, headache, hemiparesis	5 years	Eltrombopag (50 mg)	4 days	No	95,000	Negative	Superior sagittal and transverse sinuses	Enoxaparin. Clinical improvement
Rasheed et al. [10], 2020, Qatar	F/39	Headache, nausea, vomiting, neck stiffness	Chronic	Eltrombopag (irregular use)	Irregular	No	32,000	ANA+, weakly positive IgM anticardiolipin	Transverse sinuses and left sigmoid and posterior superior sagittal sinuses	Heparin followed by warfarin. Improvement
Khattak et al. [11], 2021, Qatar	F/36	Headache, left-sided weakness	11 years	Eltrombopag (75 mg)	9 months	No	N/A	Negative	Partial superior sagittal sinus and right cortical parietal vein	Enoxaparin followed by warfarin. Clinical improvement
Teekaput et al. [12], 2022, Thailand	F/29	Headache, blurred vision, nausea	4 months	Eltrombopag (50 mg)	1 month	No	212,000	Negative	Left transverse and sigmoid sinuses and left internal jugular vein	Enoxaparin followed by edoxaban. Improvement
Teekaput et al. [12], 2022, Thailand	M/75	Headache, altered consciousness	3 months	Eltrombopag (50 mg)	1 month	No	81,000	Negative	Anterior superior sagittal sinus and left transverse-sigmoid junction	IV heparin. Poor outcome; palliative management
Fares et al. [13], 2023, Morocco	F/49	Headache, seizures, altered consciousness	1 year	Eltrombopag (50 mg)	10 days	N/A	48,000	Negative	Left transverse and superior sagittal sinuses	LMWH followed by rivaroxaban. Clinical recovery
Mishra et al. [14], 2024, India	F/27	Severe headache, projectile vomiting	~2 years	Eltrombopag (75 mg)	4 months	No	291,000	Negative (APLA–)	Left sigmoid sinus and extensive thrombosis	Enoxaparin. Death
Lin et al. [15], 2025, Taiwan	F/20	Headache, nausea, vomiting	1 year and 8 months	Eltrombopag (50 mg)	5 days	No	589,000	Negative	Vein of Galen and straight, right transverse, and sigmoid sinuses	Enoxaparin followed by edoxaban. Recurrence after re-exposure
Mathews et al. [16], 2025, India	F/32	Headache, vomiting, left ptosis	Recent	Eltrombopag (50 mg)	1 month	No	624,000	N/A	Left transverse sinus	Heparin followed by acenocoumarol. Recovery
Raja et al. [17], 2025, Sri Lanka	F/18	Headache, photophobia, vomiting	Chronic	Eltrombopag (25 mg)	2 weeks	No	123,000	Negative	Superior sagittal and sigmoid sinuses	LMWH followed by warfarin. Resolution
Berrocal et al. (present case), 2025, Colombia	F/33	Progressive headache, seizures	Chronic	Romiplostim (weekly)	5 years	No	220,000	ANA 1:640 (positive)	Left sigmoid sinus and vein of Labbé	Enoxaparin followed by apixaban. Asymptomatic at discharge

One proposed mechanism underlying the paradoxical occurrence of thrombosis in patients with ITP is the increase in platelet microparticles (PMPs). These submicrometric vesicles (<0.5 μm), derived from platelet membranes and not detected by conventional platelet counts, are generated primarily during platelet activation. Several studies have demonstrated elevated PMP levels in patients with ITP compared to healthy individuals [10]. While these microparticles may have a protective role against bleeding by contributing to hemostasis, high concentrations promote thrombin generation and thrombus formation.

Additionally, treatments used in ITP may further contribute to a prothrombotic state. Intravenous immunoglobulins (IVIG), for instance, have been associated with increased blood viscosity, enhanced thrombin generation, and endothelial alterations, including cerebral vasospasm. In the context of TPO-RA therapy, such as romiplostim, there are increased platelet reactivity, enhanced platelet-monocyte aggregate formation, the upregulation of glycoprotein VI (GPVI), and increased adhesion under flow conditions, all previously associated with thrombosis [18].

Moreover, accelerated clot formation and increased levels of plasminogen activator inhibitor-1 (PAI-1) contribute to the formation of clots that are more resistant to fibrinolysis, favoring thrombus persistence [19]. Increased procoagulant microparticles and phosphatidylserine exposure on platelet surfaces following TPO-RA-induced apoptosis further enhance thrombin generation, contributing to a procoagulant state [20].

Clinically, LVT is characterized by focal neurological symptoms related to the temporal lobe territory, including aphasia, confusion, and contralateral motor deficits, particularly in the presence of venous infarction [21]. Digital agnosia may occur due to the involvement of the left angular gyrus (posterior parietotemporal junction), whereas asomatognosia is classically associated with lesions in the non-dominant parietal lobe, particularly the supramarginal gyrus and adjacent regions of the right hemisphere.

In our patient, evidence of prior partially recanalized superior sagittal sinus thrombosis with residual siderosis may plausibly explain the presence of asomatognosia. Headache is also a common presenting symptom, reported in up to 86% of cases, typically acute or subacute in onset and often preceding focal neurological deficits. Seizures are observed in approximately 26.6% of cases and may represent the initial manifestation of cortical venous involvement [22].

In this context, a high-titer ANA (1:640, fine speckled pattern AC-4) in a patient with ITP and cerebral thrombosis suggests an underlying autoimmune diathesis and warrants further evaluation, although it is not diagnostic of a defined connective tissue disease (CTD). ANA positivity is not uncommon in ITP and has been associated with an increased risk of progression to CTDs, particularly systemic lupus erythematosus (SLE), with studies reporting a significantly higher risk in ANA-positive patients (adjusted HR: 6.15; RR: up to 26.29) [23,24]. However, the absence of ENA antibodies reduces the likelihood of classical ENA-mediated CTDs, and high-titer ANA may occur in ITP without clinical progression. Therefore, its interpretation should be contextual, integrating clinical features, additional autoantibodies, and complement levels, rather than relying on ANA titers alone.

First-line treatment for ITP is based on corticosteroids, including prednisone and/or high-dose dexamethasone, with recommended duration limited to a maximum of six weeks [25]. Second-line therapy is considered in patients with inadequate response after three months or relapse during steroid tapering. TPO-RAs, such as romiplostim, eltrombopag, and avatrombopag, are preferred over splenectomy due to their sustained efficacy and favorable long-term hematologic response.

In patients who develop thrombosis associated with TPO-RAs, current clinical practice recommends the discontinuation of the agent, given the increased risk of both venous and arterial thrombotic events, even in the presence of normal or low platelet counts. Cohort studies have shown a higher recurrence rate in patients who continue TPO-RA therapy after the initial thrombotic event [26].

Anticoagulation remains the cornerstone of treatment, typically with low-molecular-weight heparin or direct oral anticoagulants, provided that platelet counts and bleeding risk allow [27]. Full-dose anticoagulation is generally recommended when platelet counts exceed 50,000/μL; otherwise, dose adjustments or alternative strategies may be considered based on thrombotic severity and bleeding risk.

In our patient, anticoagulation with apixaban and corticosteroid therapy (prednisolone) was initiated, and the TPO-RA was discontinued following the thrombotic event. Definitive management of chronic ITP was deferred for outpatient evaluation, with consideration of transition to immunosuppressive therapy such as rituximab.

This report has several limitations. A definitive causal relationship between romiplostim and the thrombotic event cannot be established; however, a clear temporal association supported by biological plausibility was identified. Detailed information regarding prior therapies, long-term follow-up, and longitudinal platelet count trends was limited, as the patient had been managed at an external institution and presented in an emergency setting. Moreover, specific biomarkers of platelet or endothelial activation were not measured, precluding an objective assessment of the prothrombotic state. Additionally, the antiphospholipid antibody profile was incomplete, and at discharge, the patient was referred to hematology for ambulatory follow-up and the completion of the diagnostic workup. Finally, the presence of high-titer ANA raises the possibility of an underlying autoimmune condition, which cannot be entirely excluded as a contributing factor.

The strengths of this report include a detailed clinical and radiological characterization, a comprehensive exclusion of alternative etiologies, and a clear temporal association with long-term romiplostim use, supported by biological plausibility. It also contributes to the limited literature on TPO-RA-associated CVT. Furthermore, it highlights clinically relevant features that may aid in the early recognition of CVT in patients with ITP, particularly those receiving TPO-RAs, and provides practical insights for diagnostic evaluation and management in this context.

## Conclusions

This case highlights the importance of considering CVT in patients with ITP who present with new-onset headache, focal neurological deficits, or seizures, particularly in the presence of additional potential prothrombotic factors such as TPO-RA therapy.

Early suspicion should prompt timely neuroimaging, including venous studies, to allow accurate diagnosis. In such cases, discontinuation of the suspected agent and initiation of appropriate anticoagulation, when clinically feasible, are key components of management.

This report underscores the need for increased clinical awareness of a potentially multifactorial thrombotic risk in patients with ITP and highlights the importance of individualized risk assessment and careful monitoring during treatment.

## References

[1] Fan Y, Yu J, Chen H (2020). Chinese Stroke Association guidelines for clinical management of cerebrovascular disorders: executive summary and 2019 update of clinical management of cerebral venous sinus thrombosis. Stroke Vasc Neurol.

[2] Appaji AC, Mohan M, Kulkarni R, Kulkarni RN (2017). Anatomy of the vein of Labbé: a cadaveric study. Int J Anat Res.

[3] Boukobza M, Crassard I, Bousser MG, Chabriat H (2020). Labbé vein thrombosis. Neuroradiology.

[4] Dmytriw AA, Song JS, Yu E, Poon CS (2018). Cerebral venous thrombosis: state of the art diagnosis and management. Neuroradiology.

[5] Schulman S, Makatsariya A, Khizroeva J, Bitsadze V, Kapanadze D (2024). The basic principles of pathophysiology of venous thrombosis. Int J Mol Sci.

[6] Kang BE, Zhang S, Lesmana H, Dungan J, Reynolds K, Guha S, Best H (2025). Venous thromboembolism laboratory testing (factor V Leiden and factor II c.∗97G>A), 2025 revision: a technical standard of the American College of Medical Genetics and Genomics (ACMG). Genet Med.

[7] Mulla CM, Rashidi A, Levitov AB (2014). Extensive cerebral venous sinus thrombosis following a dose increase in eltrombopag in a patient with idiopathic thrombocytopenic purpura. Platelets.

[8] Ho P, Khan S, Crompton D, Hayes L (2015). Extensive cerebral venous sinus thrombosis after romiplostim treatment for immune thrombocytopenia (ITP) despite severe thrombocytopenia. Intern Med J.

[9] Nambiar V, Dhanya TS, Sidharthan N (2016). Cerebral venous thrombosis in refractory idiopathic thrombocytopenia treated with eltrombopag. Ann Indian Acad Neurol.

[10] Rasheed MA, Alsaud AE, Razzaq S, Fadul A, Yassin MA (2020). Cerebral venous thrombosis in a patient with immune thrombocytopenia, an apparent paradox. Case Rep Oncol.

[11] Khattak T, Mitwalli MY, Ubaid A, Shoukry A, Anjum S (2021). Eltrombopag-associated cerebral venous thrombosis. Am J Ther.

[12] Teekaput C, Nadsasarn A, Tanprawate S (2022). Cerebral venous sinus thrombosis in immune thrombocytopenia patients treated with thrombopoietin receptor agonist: case reports and literature review. Ann Med Surg (Lond).

[13] Fares S, Halloumi O, Wakrim S, Maqsodi A, Elmekkaoui A, Benlenda O, Nassik H (2023). Immune thrombocytopenia and cerebral thrombophlebitis in a patient on eltrombopag: a rare complication. Radiol Case Rep.

[14] Mishra K, Barki S, Sreen A, Saravagi G, Kumar S (2024). A rare incidence of cerebral venous thrombosis in a case of immune thrombocytopenia on eltrombopag. Ann Natl Acad Med Sci (India).

[15] Lin IW, Lim KH, Huang YC, Sung MT (2025). Eltrombopag-induced cerebral venous thrombosis: a case report and literature review. Case Rep Hematol.

[16] Mathews SR, Thomas T, Harikrishnan S, Jesurun RS (2025). Eltrombopag-induced cerebral venous sinus thrombosis. Natl J Pharmacol Ther.

[17] Raja T, Jesiah N, Mayurathan P, Kugathasan A (2025). A case of cerebral venous sinus thrombosis following eltrombopag in immune thrombocytopenia. Cureus.

[18] van Dijk WE, Brandwijk ON, Heitink-Polle KM, Schutgens RE, van Galen KP, Urbanus RT (2021). Hemostatic changes by thrombopoietin-receptor agonists in immune thrombocytopenia patients. Blood Rev.

[19] Justo Sanz R, Monzón Manzano E, Fernández Bello I (2019). Platelet apoptosis and PAI-1 are involved in the pro-coagulant state of immune thrombocytopaenia patients treated with thrombopoietin receptor agonists. Thromb Haemost.

[20] Garabet L, Ghanima W, Hellum M, Sandset PM, Bussel JB, Tran H, Henriksson CE (2020). Increased microvesicle-associated thrombin generation in patients with immune thrombocytopenia after initiation of thrombopoietin receptor agonists. Platelets.

[21] Ramsawak L, Whittam D, Till D, Poitelea M, Howlett DC (2016). Isolated thrombosis of the vein of Labbé - clinical and imaging features. J Acute Med.

[22] Ropper AH, Klein JP (2021). Cerebral venous thrombosis. N Engl J Med.

[23] DeSouza S, Angelini D (2021). Updated guidelines for immune thrombocytopenic purpura: expanded management options. Cleve Clin J Med.

[24] Liu Y, Chen S, Yang G (2021). ANA-positive primary immune thrombocytopaenia: a different clinical entity with increased risk of connective tissue diseases. Lupus Sci Med.

[25] Pamuk ON, Ali SM, Hasni S (2023). Development of systemic lupus erythematosus in patients with immune thrombocytopenic purpura: a systematic meta-analysis. Autoimmun Rev.

[26] Palandri F, Rossi E, Bartoletti D (2021). Real-world use of thrombopoietin receptor agonists in older patients with primary immune thrombocytopenia. Blood.

[27] Saposnik G, Barinagarrementeria F, Brown RD Jr (2011). Diagnosis and management of cerebral venous thrombosis: a statement for healthcare professionals from the American Heart Association/American Stroke Association. Stroke.

